# Food addiction research in India: a scoping review

**DOI:** 10.3389/fpubh.2026.1825506

**Published:** 2026-05-08

**Authors:** Asmita Jain, Manoj K. Pandey, Rithvik S. Kashyap, M. Kishor

**Affiliations:** 1 Department of Clinical Psychology, JSS Medical College and Hospital, JSS Academy of Higher Education and Research, Mysuru, India; 2 Department of Clinical Psychology, Post-Graduate Institute of Behavioral and Medical Sciences, Raipur, India; 3 Department of Clinical Psychology, JSS Medical College and Hospital, JSS Academy of Higher Education and Research, Mysuru, India; 4 Department of Psychiatry, JSS Medical College and Hospital, JSS Academy of Higher Education and Research, Mysuru, India

**Keywords:** culture, food addiction, India, review, ultra-processed food, YFAS

## Abstract

**Introduction:**

This scoping review systematically mapped the extent of research on food addiction in India. While multiple studies have examined food addiction in the Indian context, a comprehensive understanding of its psychosocial and cultural variations remains lacking. To address these gaps and provide coherence to the literature, this review synthesized the available studies and offers a consolidated perspective.

**Methods:**

Four databases were searched for studies published until 08 April 2026. All studies addressing food addiction in the Indian context were considered, and animal studies were excluded from the review. The study selection process followed a three-step search strategy. An internally developed form was used for data extraction, and two reviewers independently screened the records. Data synthesis was conducted using both descriptive and qualitative approaches to ensure comprehensive coverage of the available evidence.

**Results:**

634 records were identified, of which ten articles met the inclusion criteria for the final review. The reported prevalence of food addiction ranged from 7.3% to 44.1%, with a median prevalence of 15.3%. Seven studies used the Yale Food Addiction Scale (YFAS) or its variants, and a considerable heterogeneity in the assessment tools was observed. The most commonly endorsed symptom domain was persistent desire or repeated failure to reduce intake. Significant associations were found between food addiction and obesity (*n* = 4). However, associations with gender and age were inconsistent, diverging from global trends. Among students, academic performance, stress, and hostel environments emerged as significant and unique risk factors (*n* = 3).

**Conclusion:**

These findings highlight the need for culturally validated assessment tools to explore the psychological and cultural correlates of FA in the Indian context, as well as for nationally representative studies. Moreover, the intersection of food addiction with unique sociocultural factors, such as dietary norms, social rituals, and academic environments, warrants targeted public health interventions and the development of national guidelines, particularly given the growing burden of obesity in the country.

## 1 Introduction

Food addiction (FA) is defined as an eating behavior characterized by the excessive consumption of specific foods in a manner analogous to substance addiction (1). Although FA is not yet recognized by major diagnostic systems, such as the Diagnostic and Statistical Manual-5 (DSM-5) or International Classification of Diseases-11 (ICD-11), it has garnered increasing scholarly attention as a potential addictive disorder. A comprehensive meta-review of 52 studies on the validity of food addiction concluded that FA constitutes a unique construct (2), meeting the diagnostic criteria for both behavioral addiction (repeated engagement in behaviors despite adverse outcomes) and substance addiction (compulsive use of particular substances). In line with this, the literature indicates that highly processed foods containing sweeteners and sugar, high-sodium foods, artificially flavored foods, and foods high in carbohydrates and saturated fats activate neural pathways similar to those implicated in substance use disorders (3), suggesting a comparable dysfunction within the brain reward system to that observed in drug or alcohol addiction. Additionally, the propensity of food to lead to delayed dopamine increase through heightened glucose and sugar levels has been understood to play a role in the development of food addiction. Other mechanisms include alterations in serotonin, impaired cognitive control, impulsivity, and emotion dysregulation (4). This further supports the view that food addiction may represent a neurobiological disorder rather than a behavioral issue.

The core diagnostic criteria for food addiction proposed in the literature include the consumption of food in larger quantities or over a longer period than intended, intense preoccupation, craving, and continued use despite awareness of adverse consequences (4). These criteria were first operationalized with the development of the Yale Food Addiction Scale (YFAS) and have since been modified in subsequent versions of the scale.

Two global reviews reported a mean prevalence of 14% among non-clinical adults and 15% among children and adolescents (5, 6). The prevalence rates exceed 20% in clinical populations (5). Although region-specific prevalence estimates are scarce, a study from Latin America reported a similar prevalence of 15% in non-clinical populations (7).

Recent research has witnessed an expansion of interest in food addiction, with studies focusing on its etiology, prevalence, and associated comorbidities. Frequently identified comorbidities include eating disorders, diabetes, obesity, depression, anxiety, and stress. Socio-demographic factors, such as sex and age, are also associated with FA. Collectively, these findings illustrate the relationship between FA and mental health and diet-related disorders.

Nevertheless, the cultural context in which food addiction occurs remains insufficiently explored. Recent research suggests that cultural variations exist in food preferences and manifestations of addictive eating behaviors (8). For example, a study involving the Japanese population reported a greater preference for savory foods, such as rice and sushi, rather than sweet foods, which are frequently the focus of Western-centric food addiction research (8). This suggests that the food triggers for addictive eating are regionally dependent. Moreover, prevalence rates varied by country, with South Asian participants endorsing higher levels of food addiction than East Asian participants. These cultural variations are therefore important to consider, as it is evident that food addiction does not manifest uniformly across diverse sociocultural contexts (9).

Given this background, India is a critical setting for investigating food addiction, as it is the second largest producer and consumer of sugarcane (10). Easy access to sugar and related products, along with greater exposure to highly palatable ultra-processed foods, may foster addictive eating behaviors. Food addiction is linked to rising obesity, which is a major public health challenge in the country. Moreover, India is the only South Asian country included in global food addiction estimates, highlighting its significant research presence. However, psychosocial and cultural variations in FA in India remain poorly understood. The regional diversity of food and sociocultural norms surrounding food may shape FA development, but the exact role of these factors remains unclear.

The available literature on food addiction in India is scattered across multiple disciplines, including psychiatry, public health, nutrition, and anthropology. Furthermore, the phenomenon of food addiction has been investigated through a variety of methodological approaches, including cross-sectional surveys and qualitative studies. This disciplinary and methodological fragmentation has prevented the consolidation of a cohesive evidence base. Existing systematic reviews, which typically prioritize quantitative and clinically standardized studies, may have excluded qualitative investigations and studies using indigenous assessment tools. Thus, there are gaps in understanding how food addiction manifests in the Indian sociocultural context. Therefore, a scoping review was considered the most appropriate methodology, as it can accommodate heterogeneous study designs and identify gaps that systematic reviews may overlook. Preliminary searches confirmed that no prior systematic or scoping review has comprehensively synthesized the evidence on food addiction in India. The review thus aimed to synthesize and contextualize the existing literature on food addiction in the Indian context, with particular attention to prevalence, assessment methods, and psychosocial and cultural variations.

Accordingly, the following research question was formulated to guide the review and ensure alignment with the objective:

What does the existing literature report regarding food addiction in the Indian population, specifically in terms of prevalence, assessment methods, and psychosocial and cultural variations?

## 2 Methods

The methodology and reporting framework of this review adhered to the Preferred Reporting Items for Systematic Reviews and Meta-Analyses extension for Scoping Reviews (PRISMA-ScR) and the Joanna Briggs Institute (JBI) guidelines (11, 12). An internally developed protocol was created prior to the study but was not registered.

### 2.1 Inclusion and exclusion criteria

The Inclusion and Exclusion criteria were developed using the Population, Concept, and Context (PCC) framework recommended by JBI. All studies conducted on human participants of any age were included, while animal studies were excluded. With respect to the concept, all studies examining food addiction or addictive-like eating behaviors were eligible for inclusion. Studies focusing exclusively on fast-food consumption, dietary patterns, binge eating, or specific foods such as sugar addiction were excluded. Only research conducted in India was included. Studies conducted on LMICs (Low- and Middle-Income Countries) without specific data on India were excluded. No restrictions were placed on publication timing to capture all available research in this area. Additionally, all study designs were included in the review.

### 2.2 Types of sources

This scoping review considered analytical observational studies, including prospective and retrospective cohort studies, case–control studies, and analytical cross-sectional studies, as well as descriptive observational study designs, including case series, individual case reports, and descriptive cross-sectional studies. Review articles and meta-analyses were also considered for inclusion.

Qualitative studies focused on qualitative data, including but not limited to phenomenology, grounded theory, ethnography, qualitative description, action research, and feminist research were included. Text and opinion papers were also considered for inclusion in this scoping review.

### 2.3 Search strategy

The 3-step search strategy recommended by JBI was used. An initial search of the PubMed database was conducted to identify articles on this topic. The terms used in the abstracts and titles of these articles were then used to develop the full search strategy to be applied across all databases with appropriate modifications. The reference lists of the included articles were screened for additional articles.

The following electronic databases were searched from inception to 17 December 2026, and updated on 08 April 2026: PubMed, Scopus, and Web of Science.

The following search strategy was applied to all databases:

Food Addiction OR Addictive Eating OR YFAS OR Yale Food Addiction Scale AND India*.

Additionally, Google Scholar results (organized by relevance) were included in the review to capture non-indexed Indian articles as part of grey literature. This is consistent with prior scoping reviews, which recommend limiting the results to the first 200 hits for feasibility (13)

### 2.4 Search selection

All records retrieved from the database searches were imported into Zotero and de-duplicated. Two reviewers independently screened the titles and abstracts according to the inclusion criteria. Full-text screening was subsequently performed on potentially relevant articles. Articles excluded at the full-text stage were excluded for the following reasons: non-Indian context, animal studies, or content unrelated to the concept of addictive eating or food addiction. The reasons for this are also presented in the PRISMA flow diagram. Disagreements regarding article selection were resolved through discussions among the reviewers.

The included studies were not appraised for methodological quality, as quality assessment is not mandatory for scoping reviews.

### 2.5 Data extraction

Data were charted using a predefined extraction form developed by the authors. The extracted information included the author, year of publication, state, study design, sample characteristics, setting, assessment tools, and key findings. Key findings were operationalized to include prevalence estimates, symptom endorsement patterns, and statistically significant correlates reported in each study. The form was piloted across three studies and subsequently modified to incorporate the themes that emerged from the review. The thematic categories for data extraction included physical, dietary, socio-environmental, and psychological correlates; the most endorsed YFAS domain; and socio-demographic correlates such as gender and age. Data extraction was completed by one reviewer and verified by a second reviewer to ensure accuracy.

### 2.6 Data synthesis

The data were quantitatively and qualitatively synthesized to map the prevalence and key characteristics of food addiction in India. The findings were organized thematically according to study characteristics, prevalence estimates, assessments used, and reported clinical and cultural correlates of food addiction.

## 3 Results

**Figure 1:**
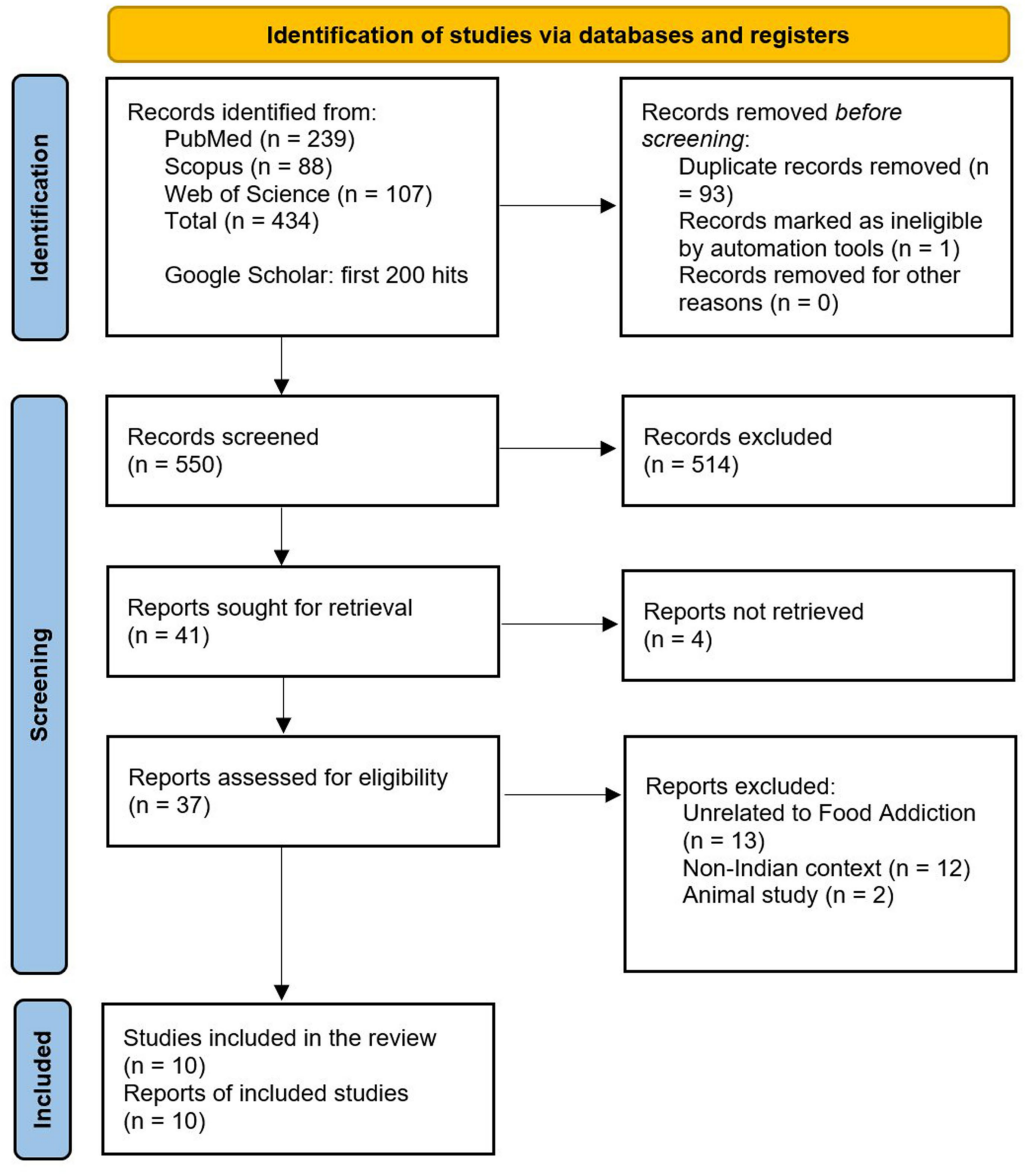
PRISMA flow diagram of the study selection process.

The study selection process is summarized in the PRISMA flow diagram (Figure 1).

### 3.1 Search results

Searches across the three databases yielded 434 records, supplemented by the first 200 results from Google Scholar, for a total of 634 records. Following deduplication, which removed 94 records, the remaining records were screened by two reviewers based on the title and abstract, after which 41 articles were retained for full-text retrieval.

Two articles could not be retrieved. The remaining 37 articles were screened against the inclusion criteria: 12 were excluded because of a non-Indian context, 2 because they were animal studies, and 13 because they were unrelated to FA. Finally, ten articles were included in the review. Of the ten articles, 1 study used EAT-26 to assess FA, which is typically used to assess disordered eating. However, this article was retained because the concept the authors claimed to measure was FA.

### 3.2 Study characteristics

The characteristics of the included studies are shown in Table 1. The ten studies were published between 2018 and 2025 and were predominantly cross-sectional, with one commentary and one mixed-methods study. Most studies were conducted with adult samples; only two studies involved adolescents. The age range of the included studies was 10–50 years. No study examining FA in the older population was identified.

**Table 1:** Characteristics of included studies.

S.No	Authors	Year	State	Scale	Design	Duration	Participant characteristics	Setting	Key findings
1	Ramesh Masthi et al. (14)	2020	KA	Junk Food Addiction Scale	Cross-sectional	Jan–Mar 2019	500, males and females aged 18–50	Urban community	16% FA Prevalence, 79% belonging to the age group of 21–30
2	Sukesh et al. (15)	2024	KA	YFAS	Cross-sectional	Aug–Oct 2022	480, males and females aged 18–25	Urban and rural	14% Prevalence, FA was significantly associated with obesity
3	Vasgare et al. (16)	2025	MH	YFAS-2.0	Cross-sectional	Jun–Aug 2023	354, males and females aged 18–25 (Mean Age of 20.99)	Online /community	11.3% Prevalence, FA associated with physical activity, sleep, anxiety, depression, BMI, dietary habits, age, education, and SES
4	Ghosh et al. (17)	2021	India	YFAS	Cross-sectional	Oct 2020 (5 days)	376, males and females aged 18+	Online/ Community	13.3% FA prevalence, associated with higher weight and BMI
5	Das et al. (18)	2021	India	YFAS and interviews	Mixed-methods	Mar–May 2020	150 males and females aged 18–30	Online/ Community	44.1% prevalence. Lockdown altered cravings & access, only 18% ordered food online
6	Tserne et al. (19)	2021	MZ	YFAS/YFAS-C	Cross-sectional	Jun 2018–Oct 2019	864, males and females, Grade 9–12, and University students	Schools & university	14.6% prevalence, high academic performance associated with higher odds of FA
7	Rai et al. (20)	2022	KA	EAT (Eating Attitude Test)	Clinical cross-sectional	Sep 2017–Nov 2018	150 OCD, 131Controls, Males and Females aged 18–60	Psychiatric clinic	FA was not significantly more prevalent among OCD patients compared to healthy controls (7.3% vs. 8.4%)
8	Wanjari et al. (21)	2025	India	NA	Commentary	NA	NA	Academic	Calls for Indian research on medical students as they are more at risk
9	Wiedemann et al. (22)	2018	India	YFAS	Cross-sectional online survey	Not reported	415 males and females aged ≥21 years (mean 32.0 ± 7.9 yrs)	Online/community	32.5% met YFAS clinical threshold; FA associated with binge eating, depression, poorer quality of life
10	Gupta et al. (23)	2024	India	YFAS	Cross-sectional	May–July 2023	103 males and females aged 10–19 years	Online/community	33.98% (39%) prevalence; FA positively correlated with perceived stress and negatively with self-esteem

KA stands for Karnataka, MH stands for Maharashtra, MZ stands for Mizoram, FA stands for Food Addiction, YFAS stands for Yale Food Addiction Scale, OCD stands for Obsessive Compulsive Disorder.

Geographically, three studies were conducted in the southern state of Karnataka, India. Other studies were conducted in Maharashtra (*n* = 1) and Mizoram (*n* = 1); five studies reported national samples or did not specify the state. The data collection settings included urban and rural communities, academic institutions, schools, hospitals, psychiatric clinics, and online platforms (see Table 1). One study involved a clinical population of individuals diagnosed with obsessive-compulsive disorder; however, the remaining studies focused on non-clinical populations.

### 3.3 Prevalence of food addiction

The prevalence estimates across all included studies are presented in Table 1.

90% of the studies (*n* = 9) reported prevalence estimates for FA, ranging from 7.3 to 44.1%. The lowest reported estimate (7.3%) was derived from a study using the Eating Attitudes Test (EAT-26), which assesses disordered eating. Therefore, this study was considered incomparable and excluded from the summary estimates. Due to the heterogeneity of the assessment tools and study populations, a pooled prevalence could not be calculated. Instead, the median prevalence across the eight studies was estimated to be 15.3%, with an interquartile range of 13.65–33.24%. Prevalence estimates above 30% were reported in only three studies.

### 3.4 Assessment of food addiction

All cross-sectional studies used validated assessment tools, and translated versions were employed where applicable. The tools used in the included studies are listed in Table 1.

Food addiction was assessed using the Yale Food Addiction Scale or its variants in 70% of the literature (*n* = 7). These included the original YFAS (*n* = 6) and the YFAS 2.0 (*n* = 1). The YFAS was translated into Hindi, Marathi, and Kannada in 30% of the studies (*n* = 3). However, validation of the translated or indigenously developed scale in the local context was not reported in these studies. The other assessment tools used included the EAT-26 and a self-developed 16-item junk food addiction scale. The internal reliability of the latter was high (0.91), but its convergent validity was not reported.

Across studies, the most frequently and consistently endorsed symptom domain on the YFAS was ‘persistent desire and repeated unsuccessful attempts to cut down or control food intake’, followed by tolerance-related symptoms. The endorsement of this domain was observed in 78.4 to 95% of the participants (*n* = 4).

Severity profiles were varied, with 20% of the literature (*n* = 2) reporting mild food addiction as the most frequently observed classification. In contrast, only 10% of the evidence (*n* = 1) reported a higher occurrence of moderate FA than mild FA.

### 3.5 Gender and age

Gender differences in food addiction were largely absent across the included studies. In 20% of the literature (*n* = 2), the data showed a higher prevalence or endorsement of FA in women than in men, but the difference was not statistically significant. Higher odds of food addiction were observed among female university students in Mizoram, but the same was not observed among school-going adolescents.

Age was examined in relation to food addiction across several included studies, with mixed results. 50% (*n* = 5) of the literature was focused on adolescents or young adults, with age ranges commonly spanning 17–25 years. 40% of the studies (*n* = 4) included broader adult samples with age ranges extending to 50 years. Most studies reported no significant association between age and food addiction. In contrast, 20% of the literature (*n* = 2) reported a statistically significant association between food addiction and age. In both studies, the younger age group had a higher prevalence of FA. A third study also noted a higher prevalence of food addiction (79%) in the 21–30 age group; however, it was not statistically significant.

Overall, the data suggest that while younger populations may be more frequently studied and potentially more affected, demographic correlates of gender and age remain inconsistent across the Indian landscape.

### 3.6 Correlates of food addiction

The correlates of food addiction were examined in 70% of the literature (*n* = 7). Anthropometric correlates were the most commonly assessed. Higher Body Mass Index (BMI) or obesity was the most frequently identified physiological correlate, showing significant associations in 40% of the studies (*n* = 4), although one study reported FA prevalence among underweight participants.

The psychological correlates of FA were examined in 40% of the studies (*n* = 4). Studies reported associations of FA with depression (*n* = 2), perceived stress and self-esteem (*n* = 1), academic stress (*n* = 1), and Obsessive Compulsive Disorder (*n* = 1).

Lifestyle-related correlates were reported in 30% of the literature (*n* = 3). Physical activity was significantly associated with FA (*n* = 2), as was recreational screen time exceeding 4 h per day (*n* = 1), and smoking or alcohol consumption (*n* = 1). Additional significant lifestyle correlates included sleep duration and non-vegetarian dietary preference (*n* = 1).

Dietary correlates were also examined in 30% of the literature (*n* = 3). Specific food items showed high predictive value for FA, particularly cheese (OR 4.21), fried chicken (OR 3.92), and cake (OR 3.45). Cravings were frequently directed toward savoury foods such as biryani and fried chicken (*n* = 2). These were reportedly substituted by consumption of snacks such as wafers, instant noodles, and chips (*n* = 1). Chocolate was descriptively identified as a common trigger for FA in 30% of the literature (*n* = 3), although statistical associations were not reported for it.

Socio-environmental correlates were reported in 30% of the included studies. One study found higher odds of food addiction among students with higher academic performance, particularly at the school level. Social contexts, such as family functions, festivals, and gatherings, were reported as triggers of increased food cravings in 20% of the literature (*n* = 2). Environmental risk factors for FA among medical students were elicited qualitatively. These factors included campus food environments, academic stress, and limited institutional mental health support.

## 4 Discussion

This scoping review represents the first comprehensive effort to map the extent of research on food addiction in India. Ten relevant articles were identified, reflecting a substantial body of research on this topic. The included studies encompass commentary, quantitative, and mixed-methods approaches, thereby contributing to the review’s comprehensiveness.

### 4.1 Prevalence of FA

Prevalence of FA ranged from 7.3 to 44.1%, indicating significant heterogeneity across studies. The lowest prevalence was observed in a study using EAT-26, a scale that assesses the presence of eating disorders. Hence, this estimate of FA is unreliable.

The median prevalence of 15.3% is slightly higher than the global prevalence of 14% (5) and also higher than the rates of 1.2–7% reported in East Asian studies (24, 25). It’s similar to the prevalence of 15% in Latin America (7). The high prevalence may also be influenced by the inclusion of studies on adolescents.

Three studies reported unusually high prevalence rates of 36.1, 39, and 44.1%. These studies shared several similarities: they used online sampling, had a higher proportion of male respondents, and used YFAS. The online data collection mode may have introduced selection bias, thereby inflating the prevalence. Notably, these prevalence rates contradict those of another study conducted online with similar characteristics, which reported a much lower prevalence of 13.3%. Although it has been argued that higher prevalence reflects greater experience of FA symptoms in Indians, our synthesis of the included studies does not support the conclusion that food addiction is significantly more prevalent in India compared to the global estimate.

Across the included studies, younger participants were more frequently represented, while older adults were largely absent. This pattern of underrepresentation aligns with gaps identified in the broader international literature, where research on food addiction in children and the older population remains sparse (5), and suggests that age-related variation in food addiction is a critical, unaddressed research priority in the Indian context.

No statistically significant gender differences were seen in the prevalence rates of food addiction among the studies included in the review. This is a notable finding as it does not align with the findings from existing research in other countries, where females have been found to have a higher prevalence of food addiction than males (26). The lack of significant associations between gender and food addiction in Indian studies so far reflects cultural variations in gender and authority norms around food.

### 4.2 Cultural aspects

Three studies identified social gatherings, family functions, and religious festivals as primary environmental triggers for addictive eating, suggesting that India’s cultural emphasis on food in communal rituals constitutes a distinct risk pathway. Food, especially what is offered during festivals, is a medium for social bonding and reciprocity. Sweet foods in particular are considered symbolic of generosity and spirituality, making it hard to refuse them in these social environments (27). This cultural imperative creates risk by providing positive reinforcement for overconsumption and increasing the difficulty in reducing food consumption in social contexts. Indeed, ‘I tried and failed to cut down certain foods’ was the most endorsed symptom across all 4 studies that discussed the most endorsed item on the YFAS in their research.

### 4.3 Correlates of food addiction

Psychological, physiological, and social correlates of food addiction identified in Indian studies largely converge with those reported in international literature, including elevated BMI, depression, anxiety, stress, and screen use (28, 29). Notably, one study reported a high prevalence of FA even in underweight participants. This aligns with the argument that FA manifests across all weight classes (30). These shared correlates suggest that the neurobiological and psychological mechanisms underlying food addiction may be relatively consistent across populations, even where cultural expression differs. However, several correlates identified in Indian studies are notably context-specific.

Academic stress, hostel residence, and higher academic performance were associated with greater food addiction among students, a pattern that may reflect the high-pressure academic environments prevalent in Indian institutions and the limited access to nutritious food in residential settings. Academic stress and its relationship with FA may result from the high executive demand posed by examinations and coursework, which may limit inhibitory control. The failure of impulse control as a mechanism for the development of FA has been well documented. Additionally, college students in India are at risk of developing unhealthy food habits, such as skipping breakfast, consuming more sweetened beverages, and eating fast food. These habits likely result from late-night study habits, limited access to personal kitchens, and limited mess hours. The significant association between perceived stress and FA in a younger sample of students aged 10–19 years also points to a causal role for stress in the development of FA.

The synthesis also revealed certain food items commonly associated with FA in India. The most commonly identified food was chocolate, followed by chicken-based food such as fried chicken, KFC, and biryani. The identified foods are both savory and sweet, unlike the dominance of sweet foods in Western literature. These dietary correlates were discussed in only a handful of studies, indicating a gap in our understanding of which foods trigger FA in India. Nevertheless, this indicates that the food items that trigger FA in the Indian context vary significantly from those listed in the YFAS instrument and are consistent with findings from other countries reporting savory food cravings (8).

The well-established associations between food addiction and emotional regulation, impulsivity, and childhood trauma remain largely unexamined in the Indian context and represent a critical gap for future research.

### 4.4 Research gaps

Several research gaps were identified from the included studies. First, clinical populations remain underrepresented, with only one study examining food addiction in a sample of patients with obsessive-compulsive disorder. Clinical populations such as patients with diabetes, thyroid disorders, polycystic ovary syndrome (PCOS), and those undergoing bariatric surgery have not been studied. Three of the ten included studies were conducted in Karnataka, limiting the generalizability of findings to the diverse dietary practices of other Indian states. Although several studies reported using translated versions of the YFAS, no formal validation studies of culturally adapted YFAS translations have been conducted in India. Furthermore, only one qualitative study was identified. The psychological correlates of food addiction, including emotional regulation, impulsivity, and childhood trauma, remain insufficiently examined in the Indian context.

### 4.5 Strengths and limitations

This review provides the first comprehensive synthesis of food addiction research in India, strictly adhering to JBI and PRISMA-ScR guidelines. The review integrates quantitative findings with qualitative findings. This scoping review has several limitations. The literature search for this review was conducted up to April 2026; therefore, any new data published later are not represented in this review. Although Google Scholar was used to identify non-indexed articles, other grey literature, such as dissertations, may have been excluded. Methodological heterogeneity across the included studies limits the comparability of the results. Specifically, the inclusion of the study using EAT-26, which was not designed for FA, may affect the clinical precision of the findings.

### 4.6 Implications

The findings of this review have significant implications for public health policy and clinical practice in India. There is a need for culturally informed interventions that target risk factors, such as social gatherings and festivals. Considering a median prevalence of 15.3%, there is also a clear need for national guidelines on addressing food addiction, particularly within the context of the growing obesity epidemic. Finally, the association between food addiction, hostel life, and academic stress suggests the need for screening and therapeutic interventions for food addiction in students.

## 5 Conclusion and future directions

This scoping review synthesized the research on food addiction in India. The prevalence estimates in the included studies range from 7.3 to 44.1%, with a median of 15.3%. The literature was predominantly cross-sectional, descriptive, and focused on the non-clinical, general population. Consistent associations were identified between FA and obesity. Associations with gender and age were inconsistent and diverged from global trends. Academic stress and hostel environments emerged as unique risk factors among students, while social gatherings and festivals constituted additional context-specific risk factors.

Future research should prioritize the cultural validation of the Yale Food Addiction Scale (YFAS) or the development of indigenous assessment tools for food addiction in both clinical and non-clinical populations. Furthermore, there is a clear need for longitudinal and mixed-methods research utilizing geographically diverse samples to better understand the clinical and sociocultural evolution of food addiction in India.

## Data Availability

The original contributions presented in the study are included in the article/supplementary material, further inquiries can be directed to the corresponding author.
